# Lived experiences of older adults during the first COVID-19 lockdown: A qualitative study

**DOI:** 10.1371/journal.pone.0252101

**Published:** 2021-06-23

**Authors:** Ilaria Falvo, Maria Caiata Zufferey, Emiliano Albanese, Marta Fadda

**Affiliations:** 1 Institute of Public Health, Faculty of Biomedical Sciences, Università della Svizzera italiana, Lugano, Switzerland; 2 Department of Business Economics, Health and Social Care, University of Applied Sciences and Arts of Southern Switzerland, Manno, Switzerland; Jordan University of Science and Technology, JORDAN

## Abstract

**Background and aim:**

Public health measures used to mitigate the COVID-19 epidemic may have unintended, detrimental consequences particularly on older adults, whose voices and perspectives are often silent or silenced. The aim of this study was to explore the lived experiences of individuals aged 64 or older during the first COVID-19 lockdown.

**Methods:**

We conducted a qualitative study in a convenience sample of 19 older adults (aged 64+) living at home in the Italian-speaking region of Switzerland during the first COVID-19 lockdown, between April and May 2020. Participants varied in terms of gender, education, age, nationality, and socio-economic status. We conducted semi-structured phone interviews to elicit emotions, expectations and hopes in relation to the present situation, and the post-pandemic world. We inquired about opinions on the enforced public health measures, including those specifically targeting older adults, and on the societal portrayal of older adults.

**Findings:**

We found that the epidemic and the public health response to it had both generated a variety of resentments and a high degree of ambivalence at the individual, micro-, meso- and macro-social levels. We also found that labelling older adults as an at-risk sub-population inevitably contributed to public and self-stigmatization.

**Discussion:**

We conducted an in-depth qualitative investigation of lived experiences of older adults during the first wave of the COVID-19 pandemic in one of the most gravely hit region in Europe. Our findings on the complexity of unintended, detrimental consequences of outbreak responses on older adults have relevant implications for local adaptions of public health measures, and suggest that public health authorities should engage vulnerable sub-populations and promote bi-directional communication to inform and support communities.

## Introduction

Older adults (i.e., individuals aged 65 or older) have been disproportionately affected by the COVID-19 outbreak in several ways, leading some scholars to define it a “geriatric health emergency” [1–4]. Older age is associated with more severe courses of COVID-19, hospitalization, need of a ventilator to support breathing, intensive care, and mortality [5, 6]. Eight out of 10 COVID-19-related deaths reported in the United States have been among adults aged 65 years and older [7], and fatality rates for those over 80 years of age are up to five times higher than the population average [8]. Moreover, the public health measures enforced to mitigate the impact of the epidemic, in particular community restrictions, have inevitably contributed to social isolation and loneliness in older adults, with marked consequences on mental and physical health [9–12]. Stay-at-home restrictions and strict physical distancing have targeted older adults earlier, for longer, or more aggressively compared to younger age groups, contributing to increased anxiety and fear of illness and death [13], disruption of daily routines [14], denied or hampered access to daily care and treatment for conditions that are unrelated to COVID-19, and socioeconomic disruptions [15].

Some aids for older adults such as food, medications, and other essential deliveries have been offered at community and household levels. However, overall social support services have been not only insufficient but also inadequate to need. Moreover, support programs that targeted older adults may have short- and long-term consequences on intergenerational relations, and on how older adults are perceived and treated in society, paving the way for stigma and discrimination [16].

In southern Switzerland, the number of fatalities related to COVID-19 was significantly higher than the national average during the first wave of the pandemic [17]. The southern, Italian-speaking Canton of Ticino borders with the Lombardy region in Italy which was Europe’s epicenter during the first wave of the COVID-19 epidemic [18]. Movement of the virus between the two regions could be limited only marginally also because of the nearly seventy thousand Italian cross-border workers who commute daily to southern Switzerland to work in the health sector or in other essential services. Therefore, local authorities prompted and enforced personal, physical distancing, and special protection measures earlier and more strictly compared to the rest of Switzerland [19].

Early restrictions included schools and workplace closures, a complete lockdown of businesses, cancellation of public events, movement limitations, and special protection measures for older people considered more vulnerable and prone to more severe forms of COVID-19 because of medical conditions and physiological changes associated with age. Public health authorities also banned older adults to enter supermarkets and grocery shops, and introduced senior early shopping hours in pharmacies and other essential shops and services. However, communication to and negotiation with the public about these interventions lacked, and the impact on health and wellbeing of special protection measures has been utterly ignored.

While a growing body of literature has qualitatively explored the psychological and societal impact of the COVID-19 outbreak, age-specific data, particularly regarding older age, is still lacking [15]. Most studies have addressed the impact of COVID-19 on health-care providers, patients, caregivers and children [20–25]. However, most studies were conducted in China with very small samples. The voices and perspectives of older adults on their lived experiences during the pandemic and in relation to institutional decisions such as the lockdown, have so far gone largely unheard [26]. Despite the WHO’s call to collect qualitative data on the psychological and social implications of the current crisis on the most vulnerable populations [27], little is known about how older adults give meaning to the outbreak and its related restrictive measures.

We designed and conducted a qualitative study in the Italian-speaking Canton of Ticino, in southern Switzerland, which was severely hit during the first wave of the COVID-19 outbreak. Official statistics indicate that 80’718 older adults (aged 65 or older) were living in the Canton of Ticino as of December 31, 2019 [28]. The vast majority of the elders in Ticino are self-sufficient and in good physical health, although one out of four suffers from social isolation, which can be due to factors such as the absence of territorial roots (not having always lived in Ticino or in the same municipality), living alone, and having infrequent contacts with family members [29]. Participation in non-domestic activities in Ticino is generally low: just over a quarter of the elderly attend associations or organized groups at least once a month and less than a sixth have undertaken new leisure activities after retirement [29]. Our aim was to investigate the lived experiences of older adults in relation to the COVID-19 epidemic and the public health measures and policies, particularly the lockdown implemented in Spring 2020. Specific objectives included eliciting participants’ feelings, expectations and hopes in relation to the current situation and the post-pandemic world; their opinion on the public health measures implemented locally and targeting older adults, the actors involved, and the societal response to the outbreak; and their perceived, widespread representation of older adults during the pandemic. The present study has scientific, social and practical relevance that extends well beyond the geographical boundaries within which it has been conducted. Our results have the potential to improve our understanding of older adults’ experiences in relation to the pandemic and the related public health measures and may provide, at the same time, key insights for an ethical, efficient, and effective implementation of future policies at a global level.

## Materials and methods

### Study design

We conducted a qualitative study to explore the lived experiences of older adults living in southern Switzerland during the COVID-19 outbreak and, in particular, during the implementation of the lockdown between April and May 2020. An interpretive (hermeneutic) phenomenological approach guided our study. We collected data employing in-depth telephone interviews, using a semi-structured interview grid, in full compliance with physical distancing measures, which were in force at the time of data collection. Qualitative research is key to explore the nature of phenomena. It is, therefore, the optimal method for capturing social responses to the COVID-19 pandemic, as it allows to explore and describe how individuals make meaning and sense of health, disease and its related risk [30]. In addition, qualitative research provides rich and potentially useful information for disease outbreak response measures planning.

### Recruitment

To facilitate a fast and efficient recruitment, in light of the limitations imposed by the lockdown (e.g., in terms of advertising the study during public events), and begin data collection shortly after ethical approval, we used a readily available database of older adults from the local source population. The database comprised 22 older adults who had taken part in previous qualitative studies carried out by the research team between 2019 and 2020 and had accepted to be contacted for further qualitative or quantitative studies. The interviewer invited them by either e-mail or phone to participate in the present study. She provided information on the study aim and procedures, as well as assurance of anonymity and confidentiality. Subsequently, she asked those who accepted to participate to invite additional participants meeting our inclusion criteria. To be eligible for the study, participants had to meet the following inclusion criteria: be at least 64 years old at the time of the interview, be free from any hearing impairments, and be resident at their home in the Canton of Ticino in southern Switzerland. Participants who were living in nursing homes were excluded from the study. We decided to include individuals who were at least 64 in order to capture the experiences of individuals who would fall in the category of “older adults” soon after data collection. We recruited 16 participants through the database, and three additional participants through referral. We combined convenience and snowball sampling strategies to maximize the variance of our participants’ lived experiences and to reach a target sample of approximately 20 participants rapidly. We estimated the target sample size on the basis of prior methodological studies and on the adequacy of the data collected [31]. Once they confirmed their intention to participate in the study, participants were asked to provide a preferred date and time and were later contacted by phone for the interview.

The Ethics Committee of the Canton of Ticino issued a favorable opinion on the study (ID REQ-2020-00291). The objectives of the study and voluntary nature of participation were explained to participants both at first contact (either by phone or by e-mail) and before starting the interview over the phone. We obtained oral informed consent before each interview. To maintain confidentiality, we replaced names with numbers and removed any potentially identifying information from the transcripts. We encrypted and saved all audio recordings, transcripts, and participants’ personal data on a password-protected computer. We followed the Standards for Reporting Qualitative Research guidelines [32].

### Data collection

We conducted semi-structured, in-depth telephone interviews at a time convenient for participants, between Apr 2 and May 15, 2020. We chose this specific timeframe to capture participants’ lived experiences during the peak of the outbreak and through the duration of the implementation of the strictest measures (i.e., the general lockdown and the ban to enter supermarkets applying to older adults). After explicit consent from participants, we audio-recorded all interviews using a free call recorder smartphone application. We recorded participants’ gender, age, education, relational status, occupation, place of residence, and type of dwelling at the end of the interview.

MF and MCZ developed, in an iterative way and based on the literature and personal concomitant experiences, a semi-structured guideline (S1 Table) to ask participants open-ended questions designed to elicit their emotions, expectations, and hopes in relation to the current situation and the post-pandemic world (e.g.: “What allows you to move forward?”). The interview guideline also contained questions to explore their opinion on the public health measures implemented locally, including those targeting older adults, on the actors involved, and on the societal response to the outbreak (e.g.: “What do you think of the measures that have been enforced so far?”). Next, we investigated opinions and perceived representations that society gave of older adults during the pandemic (e.g.: “What do you think is the widespread opinion on older adults right now?”). Furthermore, we asked participants questions on their representation of the COVID-19 disease, the strategies they used to manage and reduce the risk of becoming infected, their organization of daily living, including any experienced difficulties and any perceived temporal changes, and information seeking behaviors and information needs (e.g.: “How have you organized your daily routine? Can you tell me about your typical day?”). After development, we informally piloted the interview guideline with our acquaintances to ensure face and content validity. The guideline went through small linguistic changes before it was eventually implemented. We allowed interviews to flexibly last between 16 and 120 minutes to adapt to participants preferences and circumstances. No visual stimuli were used during the interview. We started the interview by asking an open-ended question: “How are you coping with the current situation?”. Subsequently, we asked questions for clarification or elaboration of what the participants were saying or when the participant might forget or not think of some important information that they might miss. One of the probing questions, for example, would be: “What are the challenges you are experiencing in dealing with the present situation?”. All interviews were conducted by a purposely trained, female research assistant (IF) with previous experience in qualitative research and formal education in cognitive psychology. By the time of the data collection, IF had already followed four trainings in qualitative research methods at both bachelor and master levels and had conducted a small-scale, qualitative study. A more senior qualitative researcher, MF, supervised the data collection phase and held regular feedback sessions with IF shortly after the interviews. We conducted all interviews in the local language (Italian), the native language of both the interviewers and the interviewees. Debriefing sessions between the interviewer and a more experienced member of the research team (MF) took place shortly after each interview.

### Analysis

We transcribed audio-recordings verbatim within one week after each interview. Two members of the research team (IF and MF) independently conducted an inductive thematic analysis of the transcripts in the original language (Italian) following the six-stage comprehensive thematic analysis approach developed by Braun and Clarke [33]. The analysis included reading the transcripts multiple times to familiarize with the content, identifying meaningful quotes regardless of their length, labelling them under broader concepts, organizing the generated labels around more general themes, and creating relationships between them. In the last stage of the analysis process we identified and highlighted thematic tensions experienced by participants.

We used different approaches to enhance the validity and reliability of the analysis process and results. As we extracted data from the original sources, we verified their accuracy in terms of form and context with constant comparison between a senior member of the research team (MCZ) and the coders (MA and IF). We included deviant cases in the analysis and created tables and conceptual maps to organize the labels and the themes. We discussed the results multiple times in-between the different phases of the analysis. Disagreements in the interpretation of the findings were resolved through discussion and by making constant reference to the transcripts, which helped present both convergences and divergences within the themes. For each theme, IF and MF identified meaningful quotes for later translation. Subsequently, IF, MF and MCZ established which quotes would be most representative for each theme. IF translated the quotes from the original language (Italian) to English. A validation of the English translation was conducted by MF and later checked by EA. We used the qualitative research software NVivo for all analyses [34].

## Results

The sample was composed of 19 older adults. Most participants were women (n = 12), Swiss nationals (n = 14), retired (n = 18), resident in urban areas (n = 14), and had obtained a secondary school degree (n = 14). The average age was 75 (SD = 6.04; range = 64–85). Most participants reported to be living alone (n = 12), and in a flat rather than a house (n = 14). See Table 1 for participants’ sociodemographic characteristics.

**Table 1:** Socio-demographic characteristics of participants (N = 19).

Characteristic	n (%)
Gender	
Female	12 (63%)
Age	
64–70	3 (16%)
71–80	12 (63%)
81–85	3 (16%)
Nationality	
Swiss	14 (74%)
European	4 (21%)
District of residence	
Lugano	14 (74%)
Bellinzona	2 (11%)
Blenio	1 (5%)
Locarno	1 (5%)
Education	
Primary	14 (74%)
Secondary	3 (16%)
University	1 (5%)
Occupation	
Retired only	9 (47%)
Retired and volunteer	9 (47%)
Disable and volunteer	1 (5%)
Living status	
Living alone	12 (63%)
Living with partner	7 (37%)
Type of dwelling	
Flat	14 (74%)
House	5 (26%)

The table shows the socio-demographic characteristics of participants such as gender, age, nationality, district of residence, education, occupation, living status, and type of dwelling.

**Fig 1:**
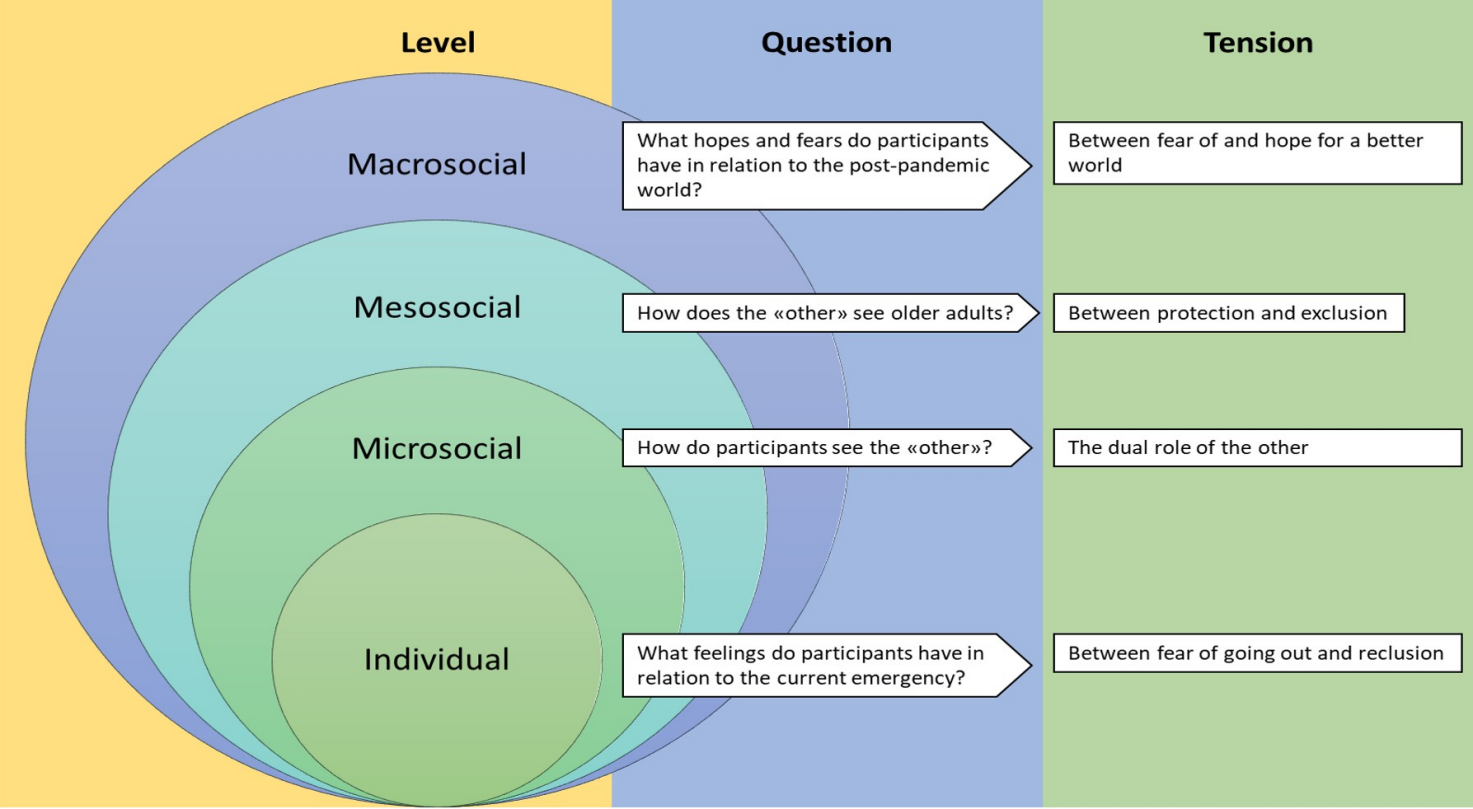
Levels, questions, and tensions. The figure shows the different levels, questions and tensions experienced by participants.

According to participants’ reports, the COVID-19 pandemic and the related mitigation measures, particularly the lockdown, generated tensions and ambivalence on the individual (concerned with emotions), micro- (concerned with the representation of the other), meso- (concerned with the widespread representation of older adults), and macro-social (concerned with future expectations) levels. At the individual level, participants reported opposite feelings, i.e., fear of going out and a feeling of reclusion at home. At the micro-social level, they reported that the other (including peers, younger people, and strangers) was seen as both a threat and a source of support. At the meso-social level, two opposite societal representations of the elderly age group emerged, i.e., the lepers vs. the victims. Finally, at the macro-social level, participants reported a combination of fear that the pandemic would change people and relationships for the worse vs. hope that it will bring wisdom and healthier connections. For a number of participants, such ambivalent feelings, beliefs, and perceptions were coexisting. Results are summarized in Fig 1.

### Impact on the individual level: Between fear of going out and a feeling of reclusion

According to most participants, the measures imposed by authorities to contain and mitigate the spread of COVID-19 and, in particular, the lockdown and the obligation to stay at home, generated a tension between opposite feelings. While participants feared leaving their house, they felt a strong sense of reclusion and isolation. When reporting these feelings, they also recognized several short- and long-term consequences of either leaving the house or being confined. The following participants reported a clear fear of leaving their house, explaining that this applies to other people too:

*I am locked inside my house and I am afraid to go out. I must stay at home because of the federal ordinance. Those who are over 65 cannot even go out to buy their groceries. Here at home, I don’t see anyone… This is how it works here. People are afraid to go out. (P2, male, 74, secondary school, living alone in a flat)*

Few participants represented the house as a place of protection from external threats. As the following participant explained, she had a mental block that impeded her to leave her house:

*Subconsciously, I had a mental block. It’s a kind of fear of that insidious stranger… And I no longer feel like going out, even for a short walk in the forest. I don’t feel like it and I only feel good if I stay at home. (P7, female, 79, secondary school, living alone in a house)*

When mentioning the short-term consequences of leaving the house, participants reported a fear of entering into contact with the virus and being infected. Participants also mentioned a number of long-term consequences of leaving the house, including the risk of being intubated or dying:

*What I really fear is the contagion, the fact of being infected and having to be intubated. That really freaks me out. Going to the intensive care and being intubated, that’s something really scary. (P9, male, 80, university, living with spouse in a flat)*

However, most participants reported a feeling of reclusion, comparing their condition to that of prisoners:

*Now, that feeling of imprisonment, that is not easy. My daughter, as a joke, told me “even prisoners need an hour of yard time”. Yes, even prisoners have the right to an hour of yard time […] I don’t know to what extent you can force people to stay at home. (P3, female, 70, secondary school, living with spouse in a flat)*

About half of the participants also anticipated that the consequences of this reclusion and lack of social contact could have a heavy impact on both their physical and mental health, including depression and loneliness:

*What sense does it make when you are not even able to see a family member? I mean, it is the saddest thing not to have the comfort of having your family next to you, to be really alone. I also understand the doctors who are dealing with this situation, it must not be easy for them. […] Finding yourself alone and not being able to even meet a friend. (P4, female, 71, secondary school, living alone in a flat)*

At the individual level, participants experienced an emotional tension in relation to their confinement at home. In the next paragraph, we present participants’ reported tensions in relation to their representation of the other.

### Impact on the micro-social level: The dual role of the other

Participants referred to a dual role of the other, when we asked them to evaluate the behavior and attitude of passers-by, acquaintances, friends, and family members in responding to the outbreak and the public health measures. For about half of participants, a potential source of transgression of COVID-19 preventive measures came from strangers (passers-by). Strangers who were not respecting COVID-19 prevention measures imposed by authorities were perceived by participants as doing the elders wrong:

*The other day I run into a lady who was wearing neither gloves nor mask, while I was wearing both. I stepped off the sidewalk and she—I think she was younger than me—she carried on her way. Because someone does not respect the rules, we are all affected. (P1, female, 72, primary school, living with spouse in a flat)*

A small number of participants reported that breaking the rules was common also among their peers. Participants expressed anger towards not only the undisciplined peers but also the institutions that were perceived as being unable to enforce the rules:

*The way they check [if people respect the rules] is disappointing. They do not check at all! Some older people… Look, I am not bad, but I would take them and punch them and then immediately take to the crematory. They are just idiots. They do not keep physical distance. One comes and walks close next to you without wearing a mask or gloves, and no one is there to set all these people straight. (P10, male, 71, secondary school, living with spouse in a house)*

When asked to provide their opinion on younger generations, most participants criticized younger people’s behaviors, arguing they showed no respect for older adults, and rarely complied to the official recommendations:

*Younger people seemed not to care; it seemed that they did not give it importance. (P11, male, 81, secondary school, living with spouse in a flat)*

*What I perceived was “They are old, anyway”. Not “Who cares?” but many young people say “Let’s go out and let’s do this and that, only old people will die”. These were the first things that you heard. (P19, female, 65, secondary school, living alone in a flat)*

On the other hand, younger people were also perceived as a source of protection. For example, three participants appreciated what young people were doing for the elders:

*Most of these young people, even here where I live, they are at home all day long. And I think they study, and read, and do other things, then they go shopping for us elders. We have younger neighbors who go shopping for us and this is actually done by many. That is why I was saying to other older people “We have to stay at home, we have to stay at home quietly, and we have all the help that we need to be able to stay at home”. (P9, male, 80, university, living with spouse in a flat)*

Of all the individuals they referred to, participants’ close ones were seen as those who always respected the rules and were genuinely interested in protecting them. This is how one participant referred to their children/nephews:

*Our daughter wants to know if we are in good health. They, both our son and daughter, check on us every day. “How are you?”, they say, and then tell us a few words like this and that to comfort us. (P9, male, 80, university, living with spouse in a flat)*

For those in stable, healthy relationships, partners represented the “best” other, who could provide company and a space for deep communication:

*My husband is two years older than me and he is also very concerned. We always help each other […] My husband and I are lucky because we have found a balance. I am not very balanced, but he balances me, and I was supporting him when he started studying. Now he is the one who supports me. (P3, female, 70, secondary school, living with spouse in a flat)*

At the micro-social level, participants referred to a tension in relation to the role of the other in responding to the outbreak and the public health measures. In the next paragraph, we present participants’ reported tensions in relation to their perceived widespread representation of older adults.

### Impact on the meso-social level: Between protection and stigmatization

From the participants’ perspective, the pandemic has exposed them to new vulnerabilities. When we asked participants what they thought was the widespread opinion on the elders, participants reported two opposite representations of the elders: on the one side, the vulnerable, i.e., those to be protected, while on the other side, the lepers that may expose others to the infection.

*Honestly, I have not yet understood if we are seen as the lepers or individuals to be protected, considering their attitude towards us… I meet, for example, a person pushing a stroller on the sidewalk, she gets off the sidewalk, which is a good thing. I would have done the same! Then she walks towards the middle of the street… I did not understand well—she was not wearing a mask either—if they are afraid that we are infected, or they do it out of respect towards us. (P4, female, 71, secondary school, living alone in a flat)*

Some participants reported to agree with their representation as the vulnerable, and felt that public health measures were meant to protect them:

*You need to protect us! This you need to do, to protect us from the outside while we protect ourselves from the inside. Yes, this must be accepted, we too must accept this situation. It’s not that we can say “I want to rebel!” No, we must accept, day by day, what we have to do, what we can do. We have to accept the current situation and believe in it… You need to respect us, and we need to respect you. (P13, female, 85, primary school, living alone in a flat)*

A minority of participants reported not to feel vulnerable but agreed with the public health restrictions imposed by authorities.

*I did not feel like a group to be protected, that is my personal opinion. But if I think about all the rest, I think it was right. However, I felt a bit deprived of my freedom. I mean, being considered at risk kind of bothers me. […] I understand why they did that, but it did bother me. (P7, female, 79, secondary school, living alone in a house)*

The majority stated that they did not feel vulnerable and did not agree with the restrictions imposed on them by authorities. As the following participant reported, older adults’ opinion was never elicited nor was it taken into consideration before implementing the measures.

*We suffered these measures, yes, we suffered them. As they said in the program that I was watching last night… There was this doctor, and he said that they have imposed these rules for our own good. Then a professor of philosophy replied that everyone decides what his or her own good is. If my own good is turn 100 years old without a penny, thank you, COVID, at some point! […] No one has ever asked us what it means for us, elders, to undergo these measures. We do have institutions, but we are not… the decision comes from the top, full stop. (P10, male, 71, secondary school, living with spouse in a house)*

These participants reported that identifying older people as an at-risk population made them feel stigmatized, discriminated, looked with suspicion, and even threatened:

*Sometimes I say “Oof! I can’t take this anymore! Today, I am going out!” But then I do not go out, because I cannot stand wearing a mask, and if I do, they look at me weirdly. […] Now we all, we are all… We look each other as is we were all infected, and we all try to stay away from each other. (P4, female, 71, secondary school, living alone in a flat)*

*I have even heard of people who have been threatened because they were out and because they were old. (P11, male, 81, secondary school, living with spouse in a flat)*

Others reported that identifying older people as an at-risk population may lead to a risk of social exclusion:

*If I have to mention one thing that was offensive, it was placing the over 65 in a position of… they cornered them! This, I did not like at all! It was better, in my opinion, to say that they could go shopping and do their stuff at their own discretion, rather than to stress so much that old people are old, that old people are at risk of infection. (P16, female, living alone in a flat)*

Others reported that being identified as an at-risk population may reinforce a representation of them as the “weak”, and eventually lead to a risk of suffering from monetary exploitation:

*Who bothers me are those who take advantage of these things, especially those who target people, right? The stronger asks the weaker “Do you want to buy this?”, “do you want to see this?” That bothers me, when someone takes advantage of these things… It seems that it is not so obvious to eradicate those things. (P6, male, 73, secondary school, living with spouse and children in a house)*

Participants who did not share their representation as the vulnerable reported the feeling they were reduced to one single, at-risk category that does not consider individual differences and thus deprives them of their own identity, generating stigmatization and victimization.

*All this commitment to safeguard the health of the over 65 has made me feel so old, as old as I have never felt before! Receiving that latter from the Canton first, and then from the municipality, that letter advising me “Dear old lady, stay at home and get your shopping delivered, etc…”, has hurt me in my active, independent, and free nature. That letter has made me dependent. (P7, female, 79, secondary school, living alone in a house)*

*I found it a bit exaggerated to start at 65 years, because 65-year-old people today, they are healthy, they are in good shape. But also at 70! I will soon be 80. I found it a bit ridiculous, I accepted it, but it has always been difficult, because a person who is 60 can be old or can be young, and the same thing at 70 and even 80. If one is healthy and active, and does many things, one can be young at 80. (P12, female, 79, secondary school, living alone in a flat)*

*They make you work until you are 64 and then, when you turn 65, they lock you in your own house! I mean, it is not like… there are people who still work at age 65 and over. They always want to increase the retirement age or whatever, and then after that… (P19, female, 65, secondary school, living alone in a flat)*

At the meso-social level, participants reported to find themselves between two opposite representations of the elders. In the next paragraph, we present participants’ reported tensions in relation to their future expectations in relation to the post-pandemic world.

### Impact on the macro-social level: Gestation of a new world

When we asked participants how they imagined the world after COVID-19, the majority replied that they were afraid that the post-COVID-19 world would be significantly different and that relationships among individuals will never be the same as they used to be. As the following two participants reported, relationships will be cold and suspicious once the virus has stopped spreading:

*[Interviewer: When you said you feel sad, what were you thinking about?] The unknown of what will come after… How we will get out of this problem… If we will still be the same… I don’t know. […] Precisely the fear of…what we will be after all this. […] We hope to go back to normality. This is our fear, but when will it end? Will it really end or not? […] They [people] will change, they will not be like they used to be. There will always be that thing. Saying “Be careful not to be like before, with all those hugs and kisses, when we used to meet each other”. I think that, after this, we will be more septic and fearful, maybe it will be different, maybe something like that. I think it will be like that, because you will always be afraid that another person, if he or she has something, can pass it to me. (P1, female, 72, primary school, living with spouse in a flat)*

*Yes, of course, it will be a big change. It won’t be the same as before, I think everything will change in my way of being when others are around. (P18, 64, female, secondary school, living alone in a flat)*

Few participants also feared that people could behave irrationally and not respect the rules once the lockdown is over:

*It will be like opening a henhouse, you know, when you open a henhouse… Have you ever seen it, in the morning? […] Well, as soon as you open a henhouse in the morning, the first thing you would see are the hens rushing to the meadow. And I am afraid that we might get to something like that, and maybe authorities will have to say “slowly, slowly!” and enforce even stricter rules. (P6, male, 73, secondary school, living with spouse and children in a house)*

Next, when we asked participants what their biggest hope for the future was, most of them referred to their hope for better, more genuine relationships among individuals. As the two following participants reported, they hoped that the pandemic would make friendships healthier, and people more supportive and less selfish:

*[I hope] we can go back to normality soon, with the hope that all this will make us better than we are and that we can finally understand the importance of life and friendship. (P4, female, 71, secondary school, living alone in a flat)*

*We are all a bit worried because we do not know what the future will be like. Will it change? Totally! And everyone will have to change their mind. Maybe we will appreciate each other a little more […] I hope that the community has learned something… specially to help others more, to be more available, to smile more, to be happy with what we have and to defend it. (P5, female, 79, secondary school, living alone in a flat)*

Few participating, rather than fearing people’s irrational behavior, held a strong hope that people will be more responsible, particularly in relation to the environment:

*I really want to live, and I hope it will end soon, for the good of all the people of the world… and I hope that we will start over, this time in a more responsible way. […] We will be much more responsible even with respect to the world that we have destroyed, the environment that we have polluted. We will do that at least at the beginning. Then maybe we will forget with time, but I think we will carry something heavy for a while. (P2, male, 74, secondary school, living alone in a flat)*

The majority reported their hope that people will be wiser, i.e., they will give a deeper meaning to their life and will be more grateful for what they have so far taken for granted, particularly their freedom of movement:

*Maybe this coronavirus will help us deepen our own meaning of life, but this depends on each of us. They always say that “The sky is darkest just before dawn”. […] This is because there is always a great hope, always! Obviously, we have to try and stay positive. (P3, female, 70, secondary school, living with spouse in a flat)*

*For sure we will really understand the value of freedom. We will give a much greater value to freedom than the one we have given it so far. (P2, male, 74, secondary school, living alone in a flat)*

*What I always tell myself is that this situation that we are experiencing should really help us. It should help us to understand and appreciate more the things that we have taken for granted. But I think that human beings are a bit like that, after a while they forgot and go back to what they used to be. I think it would be nice if it was not like that, but it’s like every time something strange happens, we all suddenly understand the meaning of life and the important things, and we all promise to change. But I have this idea that everything will go back to the way it was before, and I am afraid that will be exactly what will happen after all this. I hope that I am wrong… maybe I am too pessimistic, but I’d rather say that I was wrong afterwards. (P4, female, 71, secondary school, living alone in a flat)*

## Discussion

The goal of this study was to qualitatively explore the lived experiences of a sample of older adults living in southern Switzerland during the peak of the outbreak and, in particular, during the implementation of the Spring 2020 lockdown. We found that the epidemic and the associated protective measures had generated a variety of tensions and a high degree of ambivalence at the individual, micro-, meso-, and macro-social levels. We also found that, by recognizing all older adults as an at-risk category, the current pandemic has the potential to change the social representation of old age and reinforce ageism-based beliefs and attitudes among the population, including the elderly themselves. In the next paragraphs, we set to provide a contextualization and interpretation of our findings, and assess their implications in light of the study limitations.

Our first finding revealed a high degree of ambivalence experienced by our participants at multiple levels. Most participants experienced ambivalence intrinsically, i.e., they individually held opposite positions, beliefs and feelings. Participants feared leaving their home, yet they felt as prisoners in their own homes. Older adults saw the youth both as potential transgressors of public health measures and supportive parties for the elderly in multiple ways. Older adults felt labelled by society as both the vulnerable and the lepers. Fear and hope were the two opposite feelings they most often cited when reflecting upon a post-pandemic world. These findings resonate with a recent quantitative study that found that stress associated with the pandemic and comfort from others co-occurred in older adults [26]. The authors reported that the most commonly reported stressors were confinement/restrictions, concern for others, and isolation/loneliness, while the most commonly reported sources of joy/comfort were family/friend relationships, digital social contact, and hobbies [26]. In line with our findings, a study conducted with older adults living in nursing homes identified several tensions experienced by participants’ and resulting in a feeling of being “disconnected in a shrinking world” [35]. Next, our findings on relational ambivalence at an intergenerational level (the youth as both “friends” and “enemies” of the elderly) extends evidence from previous studies [36–38], and suggest that, compared to pre-pandemic conditions, a wider gap may emerge between young and old. This may have repercussions on exclusion and discrimination of older adults, and on intergenerational contrasts and tensions. In addition to intergenerational ambivalence, our findings also suggest that anger and frustration may be common feelings towards the same peer group. Furthermore, our results suggest that public health measures targeting specifically the 65+ population may unintentionally contribute to generate an identity crisis among the elderly. These measures may entail an identity conflict between “vulnerable to” vs. “source of contagion”.

Our second main finding reveals the dramatic consequences generated by labelling all older adults under one single “at risk” category, neglecting inter-individual differences and diversity that exist through the life course, and persist in late life, irrespective of chronological age. For most of our participants, being associated with the “fragile” was inconceivable, especially since older age today is no longer a synonymous of frailty, but is often associated with an active social life, self-development, and fulfillment. Being categorized and labelled as the “vulnerable” was recognized by participants as a source of victimization, discrimination, and exclusion. Furthermore, policies targeting older adults as a single group (including positive measures such as food deliveries and negative ones such as those imposing domestic isolation and restricted shopping hours) were perceived as reinforcing age-old stereotypes that “all older people are the same and useless” [16]. Our results suggest the potential of COVID-19 public health measures and of how these are communicated to the public to abruptly and swiftly wipe away all the efforts made in recent years to promote healthy aging [9]. Other studies found that older adults have mixed feelings of vulnerability or empowerment in relation to social supportive interventions targeting older adults (including medications and grocery deliveries). Studies found that social support can be beneficial for older adults, their mental and physical health, may contribute to reduce ageism, and improve intergenerational relations, whereas restrictive measures can have opposite effects [13]. However, social and physical distancing aimed at protecting older adults from COVID-19 infection may cause increased loneliness, depressive symptoms, health problems, and also negative stereotyping of older adults (e.g., the “helpless” or “weak”) [13]. While our results are consistent with earlier findings that some older adults reported that the representation of older adults as vulnerable reduced their sense of control and made them feel incompetent and victimized [39], they bring novelty by uncovering a potential societal risk of exploitation, discrimination and exclusion.

We conducted all interviews during the first wave of the pandemic in Southern Switzerland. A contextualization of our findings is important. The situation was characterized by heightened uncertainty, a topic repeatedly identified by the literature [40]. Furthermore, stricter measures were imposed on older adults compared to the rest of the population, and measures were in general stricter in Ticino than in the rest of Switzerland. The ambivalence experienced by participants may be understood in light of an ambivalent attitude towards behavioral anti-COVID-19 measures showed by governments, as other studies have previously found [41]. Poor communication on the reasons behind such measures and on the differences between Ticino and Italy, and the rest of Switzerland, respectively, may have further contributed to increase participants’ uncertainty. A second lens through which our results may be interpreted is offered by the field of psychology. In conditions when we are facing risk or radical uncertainty, successful anticipatory thinking is more about having a range of perspectives and critical information on how a situation is evolving, and less about having the ‘right’ narrative or making forecasts [42]. When our social system no longer seems to fit the frames, the categories, and the tools we have to understand it, as in the current pandemic, experienced ambivalence–the simultaneous experience of positive and negative emotions and thoughts–may play a positive role in managing uncertainty. Approaching ambivalence in a spirit of open curiosity rather than repressing it and polarizing to either positive or negative emotions can have positive outcomes, such as trust, adaptation, creativity, openness to and generation of new information [43, 44]. In this sense, our study contributes to the scarce literature on the impact of the lockdown on older adults and to our understanding of older adults’ coping during disasters and the ageing process, by introducing ambivalence as a possible strategy to manage uncertainty.

Our findings have both theoretical and practical implications. Our participants’ reports suggest that the concept of vulnerability adopted by authorities when imposing restrictions on older adults did not take into account the many layers of vulnerability that may develop in old age and failed to address the multidimensionality aspects of aging [45]. As a result, this concept was not shared by the majority of our respondents, who eventually identified it as a source of vulnerability itself and as leading to detrimental consequences, such as victimization, exclusion, and stigmatization. In addition, identifying one single vulnerability (being at higher risk of COVID-19) for such a diverse age group (65+) was reported to reinforce, both among respondents and society, ageism, the idea that all older people are the same and of little value for society. Our findings raise the question on how to define vulnerability. If vulnerability is defined as an identifiably increased likelihood of incurring additional or greater wrong, identifying the vulnerable and the type of special protection that they need requires that we start from the sorts of wrongs that are likely to occur and the likely degree that these wrongs will occur [46]. During the first wave of the pandemic, vulnerability has mainly been approached by identifying high risk groups, like the elders and individuals with co-morbidities. Yet vulnerability is the outcome of complex interactions of discrete risks, namely the risk of being exposed to a threat, the risk of a threat actually occurring, and the risk of lacking the defenses or resources to prevent or deal with a threat [47]. Understanding these risks and their interaction is an essential task of health policy.

### Study limitations

Some limitations of the present study are to be mentioned. The telephone was chosen as a data collection tool as it allowed to access a population that might have been difficult to reach in person or by other means. Despite studies suggest that data quality is comparable between face-to-face and telephone interviews, using this means posed some potential challenges [48–50]. The lack of an in-person contact could amplify participants’ desire to express themselves in a socially desirable manner, thus preventing participants from freely expressing their possible frustration connected to the lockdown. Furthermore, there may be challenges to interpersonal communication, specifically in the formation of trust between interviewer and subject. To mitigate both these challenges, we introduced long pauses during the interviews and adopted a familiar, warm, and understanding approach to the interview to allow participants to express their thoughts at their own pace, reduce inhibitions, and increase their confidence that their responses will remain private. A second limitation has to do with the study sampling. The fact that most participants we contacted were part of previous studies may have implications for the findings, as they may present similarities that could reduce the variability of the sample. To reduce this bias and increase variance in participants’ experiences and reports, we combined the initial sampling strategy with a snowball sampling. Finally, being this a qualitative study with a small, mostly convenience sample, results cannot be generalized to a wider population.

## Conclusions

We found a high degree of ambivalence experienced by our participants at the individual, micro-, meso-, and macro-social levels, and discovered that, by recognizing all older adults as an at-risk category, the current pandemic has the potential to change the social representation of old age and reinforce ageism-based beliefs and attitudes among the population. With a strong focus on the lived experiences of older adults in relation to the strictest public health measures thus far implemented to contain the pandemic, our results contribute to expanding the evidence of the consequences of the pandemic among this population, and will inform the adaptation of current and future outbreak response measures at a local level, aid in the development of future public health interventions, and suggest tailored messages for effective COVID-19 prevention by public health authorities. Whether, in the long-term, the current pandemic will lead to predominantly positive, predominantly negative, or ambivalent effects as those identified in this study, and whether public health measures will change the social representation of old age in the long term, are questions that longitudinal approaches to data collection have the potential to answer.

## Supporting information

S1 Table Interview grid. The table shows the main topics and subtopics addressed during the interview and the respective questions asked to participants.
(DOCX)
