# Supplementary material for: Lived experiences of older adults during the first COVID-19 lockdown: A qualitative study
Source: PLoS One. 2021 Jun 23;16(6):e0252101. doi: 10.1371/journal.pone.0252101 (PMC8221487; doi:10.1371/journal.pone.0252101)
Supplement: S1 Table — The table shows the main topics and subtopics addressed during the interview and the respective questions asked to participants. (DOCX) [file pone.0252101.s001.docx]

**S1 Table. Interview grid.** The table shows the main topics and subtopics addressed during the interview and the respective questions asked to participants.

| **(Sub)topic** | **Question(s)** |
| --- | --- |
| Feelings and expectations | How are you right now? How would you describe your current feelings? How are you coping with the current situation?  Today, what in particular annoys you/worries you/surprises you/cheers you up?  What do you expect from your family/community/politicians/institutions during this crisis? |
| Fears, hopes and resilience | What do you think of the current situation and the way it has evolved during this month? What is your biggest fear? What is your biggest hope? What gives you strength right now? What allows you to move forward? |
| Disease representation | What do you think of the disease?  Do you know anyone who was infected? How did he/she catch it? How did it go?  Have you personally contracted the disease? How did it go? |
| Risk representation | To what extent do/did you feel at risk? Why? |
| Representation of the measures | What do you think of the measures that have been enforced so far?  To what extent do you share them?  To what extent has it been hard for you to adhere to them? |
| Representation of the stakeholders | What do you think of the principal stakeholders of this crisis?  What do you think of the way the authorities have managed the current emergency?  What do you think of the experts that have provided their opinion? Which ones have stayed in your memory? |
| Risk management | What do you do, in practice, to reduce your risk of getting infected?  Have you always done this? Has anything changed? If so, how? What made it change? |
| Daily routine | Whom do you live with? How have you organized your daily routine? Can you tell me about your typical day? Who are you in contact with and how? Do you get help from anyone? If so, from whom? What help is provided to you?  What role do organizations for the elderly play in your daily life? Do they contact you? Is there anything you would like to receive from them that you are not?  If you were to judge your life now, what would you say? How is your daily life going? How is the climate in the house? |
| Difficulties of daily living (e.g., mental health, relationship conflicts, nutrition, work, stigma) | What are the difficulties you encounter today in your daily life? Over time, have they increased, stabilized or decreased? How? How do you cope with these difficulties? What strategies do you use? Who helps you? |
| Own perception | How do you feel in relation to your age? |
| Society’s perception of older adults | What do you think is the widespread opinion on older adults right now? To what extent do you share this view? |
| Perception of older adults | What is your opinion regarding older adults today? How are they behaving in the current emergency? |
| Perception of young people | What is your opinion regarding younger people today? How are they behaving in the current emergency? |
| Cross-border commuters | What is your opinion regarding cross-border commuters today? How are they behaving in the current emergency? |
| Information and information sources | Where does the information you have about what is going on come from? Did you rather seek them or receive them? What do you think of these sources? Do people close to you inform themselves differently? If so, how?  If you were to mark the most significant events that have happened in the last month on a sheet, what would you say? Why do you consider them significant?  Whom do you trust most, in relation to the current situation? |
| Vaccination attitudes and beliefs | What do you know about the development of a covid-19 vaccine? What will your position be when it will be available? Why? |
| Final remarks | Is there anything important that we have not covered? Would you like to tell us anything else? |
